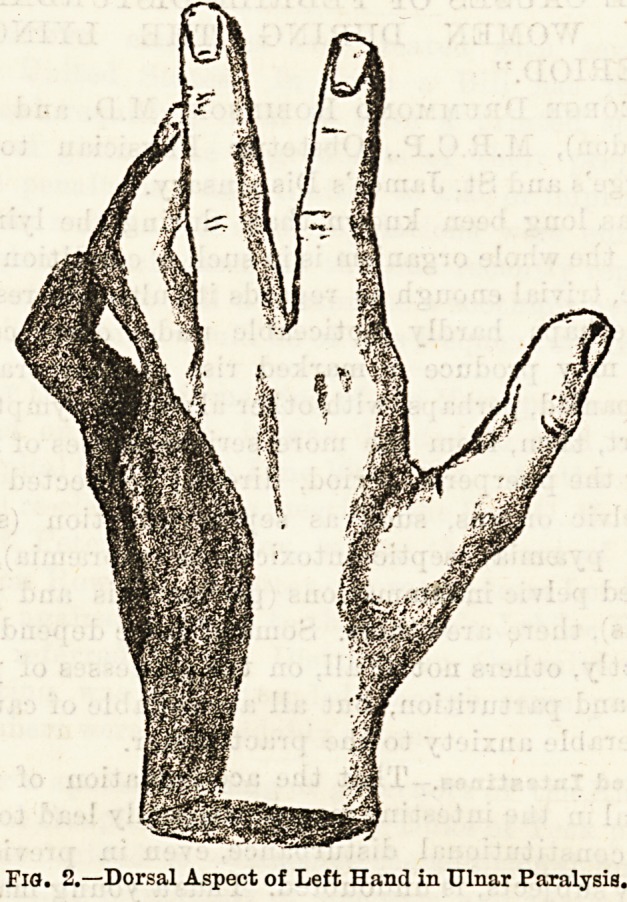# Traumatic Ulnar Nerve Paralysis.—I

**Published:** 1894-12-01

**Authors:** W. McAdam Eccles

**Affiliations:** Assistant Surgeon West London Hospital and City of London Truss Society, Assistant Demonstrator of Anatomy St. Bartholomew's Hospital, Surgeon St. Marylebone General Dispensary


### TRAUMATIC ULNAR NERVE PARALYSIS.—I.

By W. McAdam Eccles, M.B., B.S.Lond., F.R.C.S. Eng., Assistant Surgeon West London Hospital and City of London Truss Society, Assistant Demonstrator of Anatomy St. Bartholomew's Hospital, Surgeon St. Marylebone General Dispensary.

The situation of the ulnar nerve renders it peculiarly liable to injury; indeed, it is probably more frequently wounded than any other nerve in the body. Its course is comparatively superficial in the arm; but it is not here that lesions most often occur. At the elbow it lies in the well-marked groove between the olecraum process and the internal condyle, and the danger of traumatism here is almost proverbial.

Fig. 1.—Palmar Aspect of the Left Hand in Ulnar Paralysis.

Again, at the wrist it is close under the skin, and its division is very prone to take place at this spot. In the palm punctured wounds may implicate its deep branch. The division of the fibres of a nerve necessarily leads to paralysis, which may be partial or complete. In some cases of partial division sensation may remain while the motor function of the nerve is lost. The cause of traumatic ulnar paralysis is usually one or other of the following conditions: (1) Division of the nerve, partial or complete; (2) prolonged pressure upon the nerve; (3) violence applied to, with

Fig. 2.—Dorsal Aspect of Left Hand in Ulnar Paralysis.

subsequent inflammation of, the nerve; (4) a foreign body lodged in the nerve.

The results of complete division of the ulnar nerve will vary according to the exact situation at which the lesion takes place. Supposing the nerve to be divided at or above the level of the elbow joint (for the results at both spots will be the same), the whole of the distribution of the nerve will be affected, and total paralysis will occur. A lesion lower down in its course will modify the symptoms in a way which is readily grasped if the signs of total paralysis and the anatomical distribution of the nerve are borne in mind.

The signs and symptoms of complete loss of function of the ulnar nerve are very characteristic, and ought to lead to easy and prompt diagnosis. They may be divided into immediate and remote.

A patient presents himself having had an incised wound inflicted behind the internal condyle of the humerus; what are the indications that he has sustained a division of all the fibres of the ulnar nerve? The elbow-joint itself will be deprived of a part of its nerve supply, but this effect will not make itself apparent. In the forearm loss of power in the flexor carpi ulnaris and the ulnar half of the flexor digitorum profundis will result. This paralysis will be evidenced by a partial loss of flexion of the wrist, which, moreover, cannot be satisfactorily bent to the ulnar side, and by complete flexion of the ring and little fingers being impossible.

There will be no loss of sensation of the skin of the forearm. In the hand very marked changes will be found. A definite anæsthetic area can be defined embracing the region of the hypothenar eminence, and the palmar aspect of the whole of the fifth digit, together with the ulnar half of the fourth digit. On the dorsal surface the ulnar side of the hand, and the back of the little finger and the ulnar half of the ring finger will be devoid of sensation. All reaction to the stimulation disappears, the patient feeling no sensation of pain, touch, or heat and cold.

There is usually a very definite line between the non-sensitive area and the adjacent parts supplied by the median and radial nerves. This is well shown in the darkly-shaded parts in Figs. 1 and 2, which together represent the whole area of the affection, both palmar and dorsal. The patient will be unable to adduct and abduct the fingers, and also to adduct the thumb in a normal manner. This paralysis is on account of the loss of function in the three palmar and four dorsal interossei, the adductors of the thumb, and the abductor of the little finger. In addition there will be loss of the movements of the flexor brevis minimi digiti and the flexor ossis metacarpi minimi digiti. The two ulnar lumbricales and the palmaris brevis will be paralysed. The above may be taken as the typical immediate signs and symptoms which follow on high complete division of the ulnar nerve, which, if not reunited, will cause other equally significant remote symptoms. These are apparent in two ways—deformity and trophic changes. The deformity is that which is depicted in the accompanying drawings. It will be seen that the bulk of the change falls upon the fourth and fifth digits. All the first phalanges of the fingers are somewhat hyperextended, but those of the little and ring fingers very markedly so. The second and third phalanges are kept permanently flexed to a certain degree, those of the inner two fingers again being more affected than any of the others. The little finger has in addition ulnar abduction, and the whole hand is slightly displaced towards the radial side, and at the same time somewhat backwards. The cause of this deformity is by no means quite clear, yet I am convinced that it is not due alone to the *contracture* which comes on in the paralysed muscles.

When the nerve is divided high up so as to involve paralysis of the inner two tendons of the flexor profundus, the subsequent atrophic shortening of this part of the muscle undoubtedly increases the flexion of the middle and distal segments of the inner two fingers, but this flexion is present even in cases where the nerve is divided just before it enters the hand, and these portions of the fingers cannot be extended. The extension of the metacarpo-phalangeal joint is owing to the action of the extensor muscles, and in the cases of the little finger of two extensor tendons, which is unopposed by the flexing action of the interossei, and of the lumbricales in the two ulnar fingers which therefore have their first phalanges more distinctly hyperextended. The extensors appear unable to extend the middle and terminal phalanges without the help of the interossei, and I certainly think also of the lumbricales.

The ulnar adduction of the little finger is probably brought about by the want of action of the muscles of the hypothenar eminence, but I am by no means sure of this.

The trophic changes which occur are the degeneration of the paralysed muscles and certain other effects. A muscle separated from its cause of motor stimulation and its trophic centre very soon undergoes atrophy. This is more than can be accounted for by mere disuse, and in the later stages the place of the muscular tissue is taken by a mass of non-contractile fibrous tissue, which has a marked tendency to shorten, thereby producing traction and thus serious deformities.

The atrophy in the case of the muscles paralysed in ulnar nerve lesion can be very plainly seen by the loss of roundness on the ulnar side of the forearm, by the absence of the hypothenar eminence—in fact, a depression may exist in its stead—and by the hollows which exist in the region of the adductors of the thumb, and the interossei muscles. These latter are especially well seen on the dorsum of the hand.

In addition to the trophic changes in muscle tissue, the skin of the anæsthetic area becomes smooth and glossy, the hair brittle, the fingers cold and often bluish, with the nails cracked. Later changes of an osteo-arthritic character occur in the joints of the affected fingers. The secretion of sweat is decidedly diminished. In some rarer cases ulceration, or even gangrene, of the terminal phalanges may ensue.

(*To be continued.*)

## Figures and Tables

**Fig. 1. f1:**
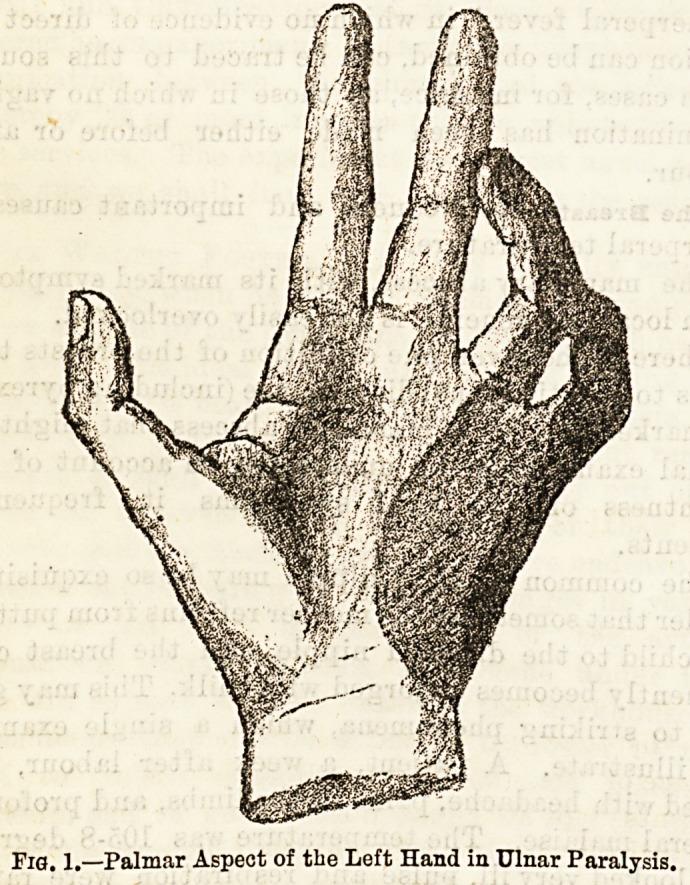


**Fig. 2. f2:**